# A Simplified, Regional Lung Ultrasound Score for Surfactant Administration in Neonatal RDS: A Prospective Observational Study

**DOI:** 10.1002/ppul.71206

**Published:** 2025-07-16

**Authors:** Francesco Raimondi, Pasquale Dolce, Claudio Veropalumbo, Enrico Sierchio, Iuri Corsini, Fabio Meneghin, Silvia Lama, Roberto Raschetti, Silvia Varano, Alessandro Perri, Luca Bonadies, Almudena Alonso Ojembarrena, Javier Rodriguez Fanjul, Rebeca Gregorio Hernandez, Lorena Rodeño Fernandez, Fiorella Migliaro, Serena Salomè, Luca Pierri, Carlo Dani, Gianluca Lista, Fabio Mosca, Virgilio Carnielli, Eugenio Baraldi, Giovanni Vento, Lucio Giordano, Manuel Sanchez Luna, Peter G. Davis, Letizia Capasso

**Affiliations:** ^1^ Division of Neonatology, Department of Translational Medical Sciences Università Federico II di Napoli Naples Italy; ^2^ Department of Translational Medical Sciences Università Federico II di Napoli Naples Italy; ^3^ Casa di Cura Pineta Grande Castelvolturno Italy; ^4^ Division of Neonatology Careggi University Hospital of Florence Florence Italy; ^5^ Neonatal Intensive Care Unit Buzzi Children's Hospital Milan Italy; ^6^ Department of Clinical Sciences and Community Health Fondazione Istituto di Ricovero e Cura a Carattere Scientifico Cà Granda Ospedale Maggiore Policlinico University of Milan Milan Italy; ^7^ Neonatal Intensive Care Unit Università Politecnica delle Marche Ancona Italy; ^8^ Fondazione Policlinico Universitario A Gemelli, Istituto di Ricovero e Cura a Carattere Scientifico Università Cattolica del Sacro Cuore Rome Italy; ^9^ Women's and Children's Health Department University of Padova Padova Italy; ^10^ Neonatal Intensive Care Unit Puerta del Mar University Cádiz Spain; ^11^ Neonatal Intensive Care Unit, Department of Paediatrics, Hospital Germans Triasi Pujol Autonomous University of Barcelona Badalona Spain; ^12^ Neonatal Intensive Care Unit Basurto University Hospital Bilbao Spain; ^13^ Neonatology Division Instituto de Investigación Sanitaria Hospital General Universitario, Gregorio Marañón, Complutense University of Madrid Madrid Spain; ^14^ The Royal Women's Hospital University of Melbourne Melbourne Victoria Australia

**Keywords:** lung ultrasound score, neonate, RDS, surfactant

## Abstract

**Background:**

A total lung ultrasound score (tLUS) is a validated tool to describe parenchymal aeration, evaluate neonatal respiratory distress syndrome (RDS) progression and guide early surfactant replacement. tLUS derives from regional scores (rLUS) from predefined ultrasound views.

**Research Question:**

This paper explores the relative contribution of rLUS to tLUS and their predictive power of surfactant need for RDS, individually and with additional variables.

**Study Design and Methods:**

This was a secondary analysis of multicenter, prospective, observational study. Preterm neonates with RDS were stabilized on nCPAP. Within 2 h of life, we calculated a tLUS (range 0–18) by summing 6 rLUS (using a 0–3 scale on midclavicular, anterior and posterior axillary line views) and the oxygen saturation/inspired oxygen fraction ratio (SatO_2_/FiO_2_). The administration of surfactant by a physician masked to the ultrasound results was used as reference test.

**Results:**

We enrolled 175 preterm infants. A midclavicular (MC) score ≥ 2 was an early marker of aeration heterogeneity. Prognostic accuracy for surfactant need was high for the left MC score (AUC: 0.86 with sensitivity 0.79 and specificity 0.90) and the right MC score (AUC 0.87 with sensitivity 0.74 and specificity 0.93; optimal Youden cut‐off = 2). A combined left + right MC score lead to an AUC: 0.90 (sensitivity 0.82. specificity 0.89; optimal Youden cut‐off = 3). A prediction model including gestational age, SatO_2_/FiO_2_ and the combined MC score had an AUC 0.95.

**Interpretation:**

rLUS are not always uniformly distributed in early RDS. The combined MC score is a simplified rapid and accurate predictor of surfactant replacement (alone or in combination with noninvasive variables) reducing stressful manipulations in first hours of life for preterm neonates.

**Abbreviations**
AUC: area under the curve
BPD: bronchopulmonary dysplasia
FiO2: inspired oxygen fraction
LUS: lung ultrasound score
MC: midclavicular
nCPAP: nasal continuous positive airways pressure
RDS: respiratory distress syndrome
rLUS: regional scores
ROC: receiver operating characteristic
SatO2/FiO2: oxygen saturation/inspired oxygen fraction ratio
tLUS: total lung ultrasound score

## 1 Introduction

Early, targeted surfactant replacement therapy reduces mortality and morbidity following moderate to severe neonatal respiratory distress syndrome (RDS) [1]. Disease progression is marked by worsening clinical and radiological signs and the need for higher concentrations of supplemental oxygen. Current guidelines recommend inspired oxygen fraction (FiO_2_) thresholds for surfactant delivery, mostly based on consensus of experts rather than on robust scientific evidence [2, 3].

The lung ultrasound score (LUS) has recently been proposed as an alternative to FiO_2_ as a criterion for surfactant replacement [4]. It uses predefined ultrasound views (usually three from each lung) which provides a total lung ultrasound score (tLUS). tLUS correlates well with oxygenation [5] and surfactant activity [6] and is a reliable marker of the clinical course of RDS [7]. A recent systematic review and meta‐analysis established that tLUS accurately predicts the need for exogenous surfactant [8]. Accuracy can be increased further by adding gestational age and the SatO_2_/FiO_2_ ratio in a three‐variable regression model [9].

The role of regional lung ultrasound scores (rLUS) in predicting disease severity is unknown. The present paper explores the rLUS distribution in a multicenter cohort of preterm babies early in the course of their RDS. The accuracy of rLUS alone and in a three‐variable model to predict the need for surfactant replacement therapy is then explored.

## 2 Study Design and Methods

This is a secondary analysis of prospectively collected data from preterm infants from 10 Italian and Spanish NICUs [9]. We included premature neonates with RDS born between 25^0^ and 33^6^ weeks of gestational age calculated from the first day of the last menstrual period and studied before their first surfactant dose. RDS was defined as the presence of intercostal and subcostal retractions with expiratory grunting shortly after birth in the presence of typical radiographic features including decreased pulmonary expansion, reticulogranular lung opacities and air bronchograms. We excluded infants who were intubated in the delivery room and those with major congenital malformations. Written parental consent was obtained and the study was approved by the Ethics Committee “Carlo Romano” at the Università Federico II di Napoli (# 1621/17). Formal approval was also obtained by the Ethics Committee of each participating center. The study was conducted in accordance with the TRIPOD statement guidelines [10] and registered in the ISRCTN Registry (number 12756197).

### 2.1 Data Collected and Aim of the Study

At enrollment, important prenatal variables were recorded. Preterm neonates were stabilized according to the local NICU protocol and those managed with CPAP were enrolled within 120 min of birth. A local investigator who was not involved in the clinical care of the patient recorded the nCPAP level in cm H_2_O, the FiO_2_ and the post‐natal age. SpO_2_ was measured by a pulse oximeter applied to the right hand and was maintained in the 90%–95% range by adjusting FiO_2_.

The investigator also performed a lung ultrasound scan on a neonate in supine position using three predefined ultrasound views (middle clavicular, anterior axillary and posterior axillary lines) on each side [11] as previously validated [12]. Each ultrasound view was assigned a score using the Brat scale (0–3). The individual scores were then added to produce a total lung ultrasound score [5, 12].

Natural surfactant (poractant alfa 200 mg/kg for the first dose and 100 mg/kg for the following doses, Chiesi Farmaceutici, Parma, Italy) was prescribed by an attending neonatologist, unaware of the results of the lung ultrasound, based on radiographic and clinical signs. Most centers delivered nCPAP using only nasal prongs, however, two used only nasal masks and one used both prongs and mask. CPAP levels were most commonly set at 6–7 cm/H_2_O, two NICUs allowed up to 8 cm/H_2_O.

The local investigator recorded the subsequent respiratory support provided and important clinical outcomes until discharge, including bronchopulmonary dysplasia (BPD) [13].

Our aim was to evaluate the rLUS performance (alone, in combination or together with other clinical variables including gestational age and the SatO_2_/FiO_2_ ratio) as predictors of later surfactant administration by a masked attending physician.

## 3 Statistical Analysis

As this is a secondary analysis of a multicenter study, no a priori power calculation was conducted specifically for this analysis. However, we have now performed a post hoc power analysis to assess the study's ability to detect a clinically relevant discriminative performance (as measured by the AUC) during model validation. Specifically, the available sample size of 71 patients in the group that received surfactant and 104 in the group that did not receive surfactant achieves 88% power to detect an Area Under the Curve (AUC) of 0.9, assuming a null hypothesis AUC of 0.8 (indicative of moderate discriminative ability), using a two‐sided z‐test at a 5% significance level.

Categorical variables were summarized as frequencies and percentages, while quantitative variables were reported as mean ± standard deviation (SD) or as median and interquartile range (IQR), as appropriate. Group comparisons were performed using one‐way ANOVA or the corresponding non‐parametric Kruskal–Wallis test for quantitative variables, and the chi‐square or Fisher's exact test for categorical variables.

A receiver operating characteristic (ROC) curve, with the corresponding Area under the Curve (AUC), was used to evaluate the discrimination performance of individual scores for early surfactant replacement. The optimal cut‐off points for each score were identified using the Youden index.

Additionally, logistic regression models were used to evaluate the discriminative ability of the individual scores, including gestational age and the SatO2/FiO2 ratio as covariates. The discriminative capacity of the models was evaluated by computing the AUC.

All statistical analysis were performed using R statistical software.

## 4 Results

A total of 175 preterm neonates were enrolled in the study (study flow chart in Supporting Information). and their demographic data are reported in Table 1.

**TABLE 1:** General characteristics of the study population (*N* = 175).

Variable	25–27 weeks (*n* = 27)	28–30 weeks (*n* = 65)	31–33 weeks (*n* = 83)	*p* value
Gestational age (days)^a^	185 ± 7.2	207 ± 5.4	226 ± 5	< 0.001
Birth weight (g)^a^	841 ± 163	1264 ± 235	1697 ± 442	< 0.001
Type of delivery (SVD)	6 (22%)	6 (9.2%)	23 (27.7%)	0.019
Chorioamnionitis	2 (7.4%)	12 (18.4%)	3 (3.6%)	0.009
Maternal hypertension	4 (14.8%)	7 (10.7%)	5 (6%)	0.322
PROM	0	2 (3%)	5 (6%)	0.453
IUGR	2 (7.4%)	8 (12.3%)	11 (13.2%)	0.777
Apgar score at 5 min ≤ 5	2 (7.4%)	0 (0%)	0 (0%)	0.023
Antenatal steroids				0.940
No	1 (3.7%)	5 (7.7%)	7 (8.4%)	
1 dose	4 (14.8%)	11 (16.9%)	13 (15.6%)	
2 doses	22 (81.5%)	49 (75.38%)	63 (75.9%)	
Age at LUS scan (minutes)^a^	102 ± 40	103 ± 39	108 ± 46	0.699
FiO2 at LUS scan^b^	0.3 (0.25–0.35)	0.25 (0.21–0.3)	0.23 (0.21–0.28)	< 0.001
LUS at scanning^b^	12 (9–12)	8 (6–12)	7 (3–10)	< 0.001
Sat O2/FiO2 at LUS scan^a^	313 ± 80	377 ± 76	394 ± 76	< 0.001
First surfactant dose (n)	20 (74%)	25 (38.4%)	26 (31.3%)	< 0.001
Late surfactant recipients (> 180 min)	8 (29.6%)	8 (12.3%)	9 (10.8%)	0.061
Age at first surfactant dose (min)^a^	248 ± 497	312 ± 401	308 ± 525	0.889
FiO2 at first surfactant dose^b^	0.38 (0.35–0.42)	0.38 (0.35–0.4)	0.33 (0.31–0.35)	0.017
Death or BPD	11 (40.7%)	10 (15.4%)	4 (4.8%)	< 0.001

Abbreviations: SVD, spontaneous vaginal delivery; IUGR, intrauterine growth retardation; BPD, bronchopulmonary dysplasia; SatO_2_/FiO_2_, oxygen saturation over inspired oxygen fraction ratio.

^a^ Data expressed as mean ± standard deviation.

^b^ Data expressed as median (interquartile range).

Table 2 describes the rLUS distribution in the three groups, categorized according to tLUS (≤ 7, 8–11, ≥ 12). For the 54 babies with tLUS ≥ 12, the pattern of a white lung (score = 2) is predominant with 4 exceptions on the middle clavicular projections showing discrete B lines (score = 1). For the infants with tLUS ≤ 7, a partial score ≥ 2 is present in 20/81 cases (24.6%) and never on the midclavicular projections. The tLUS 8–11 group reveals a gradient of partial scores, increasing as the views become more posterior.

**TABLE 2:** The regional LUS distribution divided by three LUS intervals.

	LUS ≤ 7 *n* = 81		
	LUS 0 *n* (%)	LUS 1 *n* (%)	LUS ≥ 2 *n* (%)
R MC	37 (48)	44 (52)	0
R AA	28 (37)	53 (63)	0
R PA	22 (30)	50 (58)	9 (12)
L MC	29 (40)	50 (58)	2 (2)
L AA	26 (35)	54 (63)	1 (1)
L PA	25 (32)	47 (56)	9 (11)

	LUS 8–11 *n* = 40		
	LUS 0 *n* (%)	LUS 1 *n* (%)	LUS ≥ 2 *n* (%)
R MC	2 (5)	32 (80)	6 (15)
R AA	0	17 (42.5)	23 (57.5)
R PA	0	2 (5)	38 (95)
L MC	1 (2.5)	28 (70)	11 (27.5)
L AA	0	17 (42.5)	23 (57.5)
L PA	0	6 (15)	34 (85)

	LUS ≥ 12 *n* = 54		
	LUS 0 *n* (%)	LUS 1 *n* (%)	LUS ≥ 2 *n* (%)
R MC	0	2 (4)	52 (96)
R AA	0	0	54 (100)
R PA	0	0	54 (100)
L MC	0	2 (4)	52 (96)
L AA	0	0	54 (100)
L PA	0	0	54 (100)

*Note*: There is an increase in the prevalence of LUS ≥ 2 from the MC projection through the AA to PA projection.

Abbreviations: MC, midclavicular view; AA, anterior axillary view; PA, posterior axillary view; L, left; R, right.

In general, the lower score in the midclavicular views shows a trend toward a greater aeration in the anterior regions of both lungs in the first hours of life.

**FIGURE 1:**
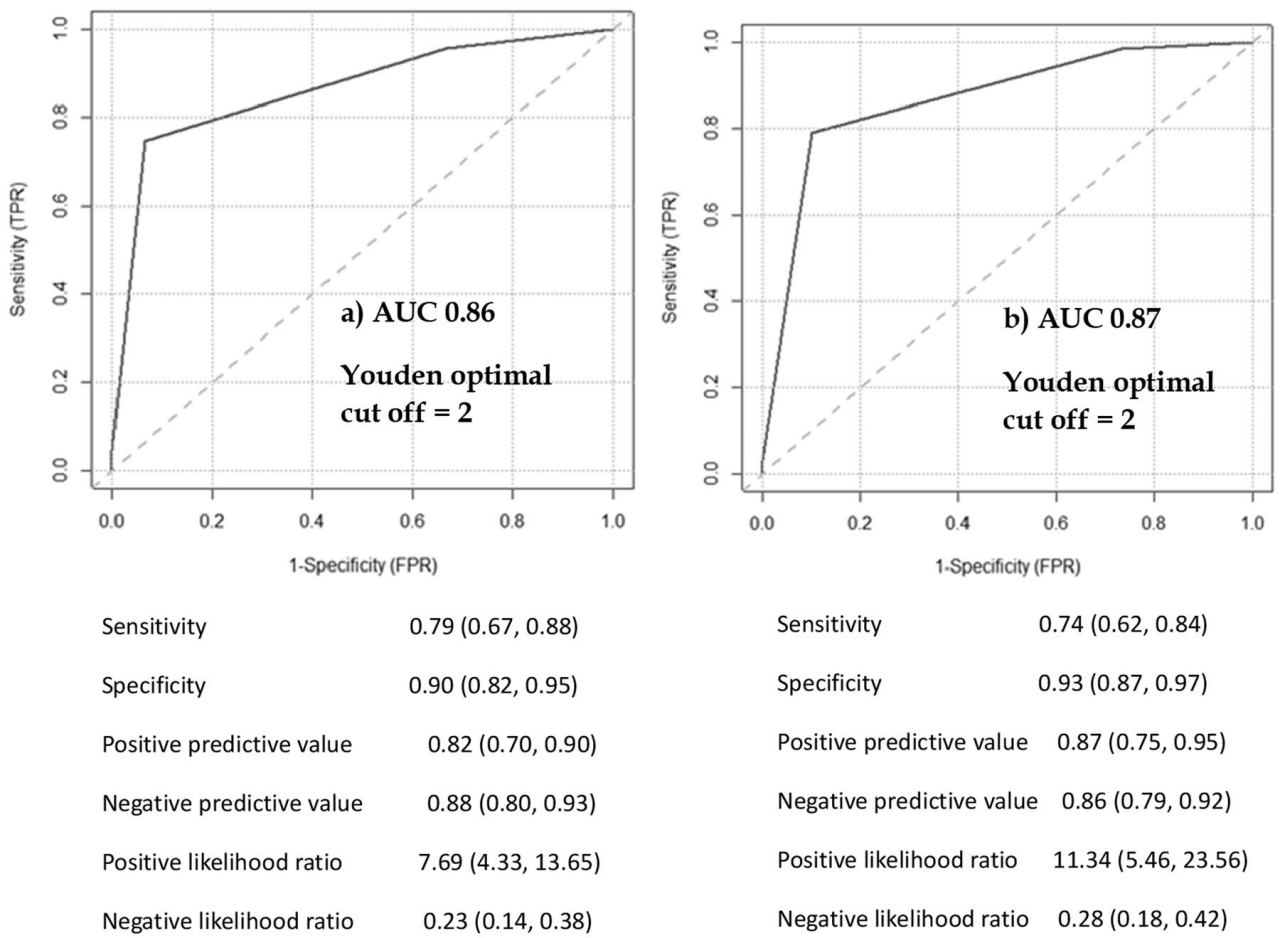
Discriminative accuracy of LUS on left (a) and right (b) MC view for later surfactant administration.

**FIGURE 2:**
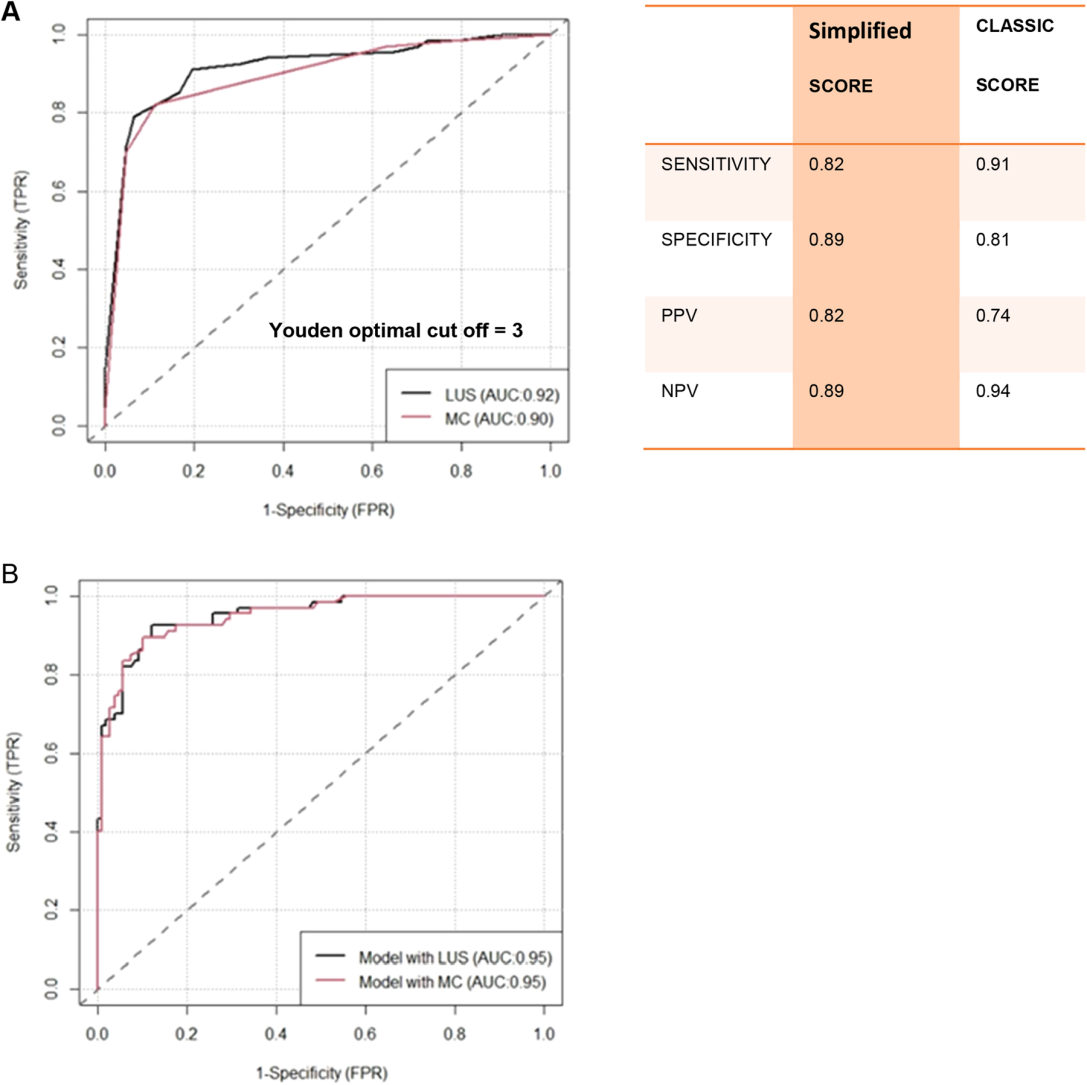
(A) Comparison between the discriminative capacity of the sum of the MC left and right scores (regional score), red line, with respect to the total LUS, black line. (B) Comparison between the discriminative capacity of the multiple regression model with the regional score as predictor, (red Line) versus the multiple regression model with total LUS, (black line). Both Multiple regressions included gestational age and the SatO_2_/FiO_2_ ratio as covariate. [Color figure can be viewed at wileyonlinelibrary.com]

Figure 1 reports the discriminative accuracy of the left (panel A) and right (panel B) middle clavicular scores for surfactant administration in the study population. The optimal Youden cut‐off = 2 for both views. As shown in Figure 2, these results can be improved if the combined middle clavicular score (left + right) is considered (panel A). For the latter the optimal Youden cut‐off = 3 of rLUS has a discriminative capacity of surfactant need with an AUC = 0.90 while the tLUS showed an AUC = 0.92.

If the combined middle clavicular score, rLUS, is considered in the regression model including gestational age and the SatO_2_/FiO_2_ ratio (panel B) as covariate, as previously described [9], its AUC = 0.95 matches that of the regression model including tLUS.

In a separate cohort of 10 infants, an experienced investigator (L.C.) took 36 ± 3 s to acquire both midclavicular scores compared to the 86 ± 4 s needed for a full scan. As this was a secondary analysis, an a priori sample power calculation on these aims was not possible. We have performed a post hoc power analysis to assess the study ability to detect a clinically relevant discriminative performance (as measured by the AUC) during model validation. Specifically, the available sample size of 67 patients in the group that received surfactant and 108 in the group that did not receive surfactant achieves 86% power to detect an Area Under the Curve (AUC) of 0.9, assuming a null hypothesis AUC of 0.8 (indicative of moderate discriminative ability), using a two‐sided z‐test at a 5% significance level.

## 5 Discussion

The distribution of partial lung ultrasound scores reveals that the anterior regions of both lungs are better aerated than the posterior regions as early as the first 2 h of life. Heterogeneity in regional aeration has previously been described using lung ultrasound in different neonatal settings. In a cohort of preterm infants, Hoshino et al showed a gravity effect on the scores of dependent areas in babies nursed prone [14]. Loi et al. recently computed the global and regional aeration heterogeneity comparing infants affected by different respiratory conditions using an extended lung ultrasound score encompassing the posterior lung areas [15]. Neonatal RDS may be distinguished from transient tachypnea, evolving BPD and neonatal acute respiratory distress on the basis of its aeration pattern [15, 16]. The early aeration changes in a ventilated preterm lamb are described by He et al. Using lung ultrasound and electrical impedance tomography in the supine animal, the authors reported that the anterior regions were better aerated soon after birth [17]. Our results are consistent with this finding and have practical implications. We used the informative power of early aeration heterogeneity to simplify a previously described predictive model of surfactant administration. Since the combined midclavicular score is a reliable predictor for surfactant replacement, the reduced data acquisition time improves patient stability and expedites personalized care. Indeed, reductions in stressful manipulations during routine care also promote healthy brain maturation of the preterm infant [18]. In a survey among healthcare professionals, Newnham et al found that doctors and nurses evaluated ultrasound scans as “moderately stressful.” It is reasonable to believe that shortening the stressor duration may benefit the infant [19].

The present paper has some limitations. The study was carried out by expert ultrasonographers, and operator experience is a potential variable. However, lung ultrasound is a readily acquired skill and the reproducibility of the LUS is high [20]. Although previous reports use the same zero to three scale for scoring the images [11], the choice of preset ultrasound views for tLUS calculation may vary. Corsini et al recently investigated three different protocols for data acquisition in a retrospective cohort of babies with RDS. The lung anterior fields were computed twice in the paper by Brat et al. [5] while Rodriguez Fanjul et al. [21] had to tilt the baby on the side to include the posterior fields. On the other hand, the choice of landmarks (midclavicular, anterior and posterior axillary lines) in the Raimondi et al. protocol [7] uniformly spans the anterior and lateral chest of the patient placed in the supine position and is slightly more accurate in predicting surfactant replacement [12]. Thus, given the early aeration heterogeneity described in this paper, the choice of ultrasound views may lead to different results.

## 6 Conclusion

During early neonatal RDS, the regional lung ultrasound scores are not always uniformly distributed. The combined midclavicular score is highly predictive of surfactant replacement and its prognostic accuracy can be improved by adding gestational age and SatO_2_/FiO_2_. The combined midclavicular score may help to reduce stressful manipulations in the first hours of life in preterm neonates.

## Author Contributions

**Francesco Raimondi:** conceptualization, funds acquisition, writing. **Pasquale Dolce:** methodology statistical analysis. **Claudio Veropalumbo:** investigation. **Enrico Sierchio:** investigation. **Iuri Corsini:** investigation, data curation. **Fabio Meneghin:** investigation, data curation. **Silvia Lama:** investigation, data curation. **Roberto Raschetti:** investigation, data curation. **Silvia Varano:** investigation. **Alessandro Perri:** investigation. **Luca Bonadies:** investigation. **Almudena Alonso Ojembarrena:** investigation, writing. **Javier Rodriguez Fanjul:** investigation. **Rebeca Gregorio Hernandez:** investigation. **Lorena Rodeno Fernandez:** investigation. **Fiorella Migliaro:** investigation. **Serena Salomè:** investigation. **Luca Pierri:** investigation. **Carlo Dani:** investigation. **Gianluca Lista:** critical reviewing. **Fabio Mosca:** methodology. **Virgilio Carnielli:** critical reviewing. **Eugenio Baraldi:** methodology. **Giovanni Vento:** investigation, methodology. **Lucio Giordano:** investigation. **Manuel Sanchez Luna:** critical reviewing. **Peter G Davis:** writing, critical reviewing. **Letizia Capasso:** conceptualization, data analysis.

## Conflicts of Interest

The authors declare no conflicts of interest.

## Supporting information

Supplementary flow chart study.

## Data Availability

The data that support the findings of this study are available from the corresponding author upon reasonable request.
