# Supplementary figures and images for: A Simplified, Regional Lung Ultrasound Score for Surfactant Administration in Neonatal RDS: A Prospective Observational Study
Source: Pediatr Pulmonol. 2025 Jul 16;60(7):e71206. doi: 10.1002/ppul.71206 (PMC12268238; doi:10.1002/ppul.71206)

## Study Flowchart

302 infants enrolled

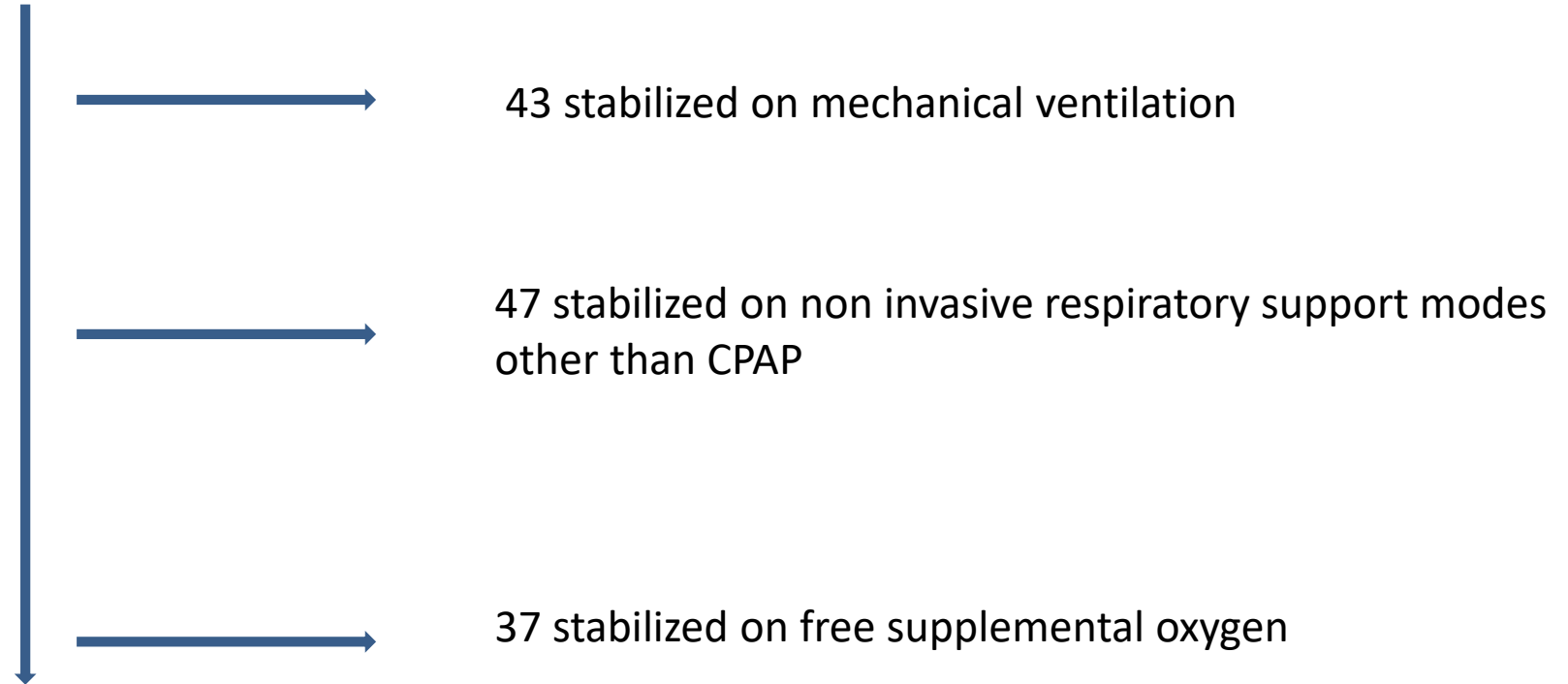

175 infants stabilized on CPAP

Supplement: Supplementary file 1 — Supplementary flow chart study. [file PPUL-60-0-s001.pdf]
